# VEGF-targeting drugs for the treatment of retinal neovascularization in diabetic retinopathy

**DOI:** 10.1080/07853890.2022.2064541

**Published:** 2022-04-22

**Authors:** Alessandro Arrigo, Emanuela Aragona, Francesco Bandello

**Affiliations:** IRCCS San Raffaele Scientific Institute, Vita-Salute San Raffaele University, Milan, Italy

**Keywords:** Diabetic retinopathy, NPDR, PDR, neovascularization, VEGF, anti-VEGF, intravitreal injection, panretinal photocoagulation

## Abstract

Diabetic retinopathy (DR) is the most common microangiopathic complication of diabetes mellitus, representing a major cause of visual impairment in developed countries. Proliferative DR (PDR) represents the last stage of this extremely complex retinal disease, characterized by the development of neovascularization induced by the abnormal production and release of vascular endothelial growth factor (VEGF). The term VEGF includes different isoforms; VEGF-A represents one of the most important pathogenic factors of DR. Anti-VEGF intravitreal therapies radically changed the outcome of DR, due to combined anti-angiogenic and anti-edematous activities. Nowadays, several anti-VEGF molecules exist, characterized by different pharmacological features and duration. With respect to PDR, although anti-VEGF treatments represented a fundamental step forward in the management of this dramatic complication, a big debate is present in the literature regarding the role of anti-VEGF as substitute of panretinal photocoagulation or if these two approaches may be used in combination. In the present review, we provided an update on VEGF isoforms and their role in DR pathogenesis, on current anti-VEGF molecules and emerging new drugs, and on the current management strategies of PDR. There is an overall agreement regarding the relative advantage provided by anti-VEGF, especially looking at the management of PDR patients requiring vitrectomy, with respect to laser. Based on the current data, laser approaches might be avoided when a perfectly planned anti-VEGF therapeutic strategy can be adopted. Conversely, laser treatment may have a role for those patients unable to guarantee enough compliance to anti-VEGF injections.

**Key messages**
- VEGF increased production, stimulated by retinal hypoperfusion and ischaemia, is a major pathogenic factor of neovascular complication onset in diabetic retinopathy and of DR stages progression.
- Nowadays, several anti-VEGF molecules are available in clinical practice and other molecules are currently under investigation. Each anti-VEGF molecule is characterized by different targets and may interact with multiple biochemical pathways within the eye.
- All the data agreed in considering anti-VEGF molecules as a first line choice for the management of diabetic retinopathy. Laser treatments may have a role in selected advanced cases and for those patients unable to guarantee enough compliance to intravitreal treatments schemes.

## Introduction

Vascular endothelial growth factor (VEGF) is one of the most important mediators involved in the pathogenesis of diabetic retinopathy (DR). The abnormal production and release of VEGF induces vascular endothelial cell proliferation and migration and increased vascular permeability [1]. VEGF is involved in the pathogenesis of DR-related complications, such as diabetic macular edoema (DME); furthermore, it represents a fundamental mediator for the development of retinal neovascularization leading to the possible onset of vitreous haemorrhage and tractional retinal detachment [2]. The term VEGF includes different isoforms, namely VEGF-A, VEGF-B, VEGF-C, VEGF-D, VEGF-E, VEGF-F and PGF (placental growth factor), the first one representing one of the most important pathogenic factors of DR. The introduction of anti-VEGF intravitreal therapies radically changed the course of DR and patients’ outcome, having a remarkable impact on the incidence of legal blindness. Nowadays, several anti-VEGF molecules exist, acting on different metabolic pathways and VEGF isoforms. The usage of anti-VEGF as first line treatment provided undoubted benefits for the management of DME; on the other side, the treatment of the proliferative form is quite controversial. Indeed, a big debate is still present in the literature regarding the role of laser approaches, administered through different strategies (prompt, deferred, etc.) in the anti-VEGF era.

In the present review, we provided an overall description of VEGF-targeting drugs and the acting mechanisms against the development of retinal neovascularization in DR.

## Methods

We used keywords to explore all English language human subject articles in the MEDLINE library. The keywords included the following: diabetic retinopathy, exudation, diabetic macular edoema, vascular endothelial growth factor, VEGF, anti-VEGF, intravitreal injections, neovascularization, ischaemia. All the references were carefully examined by two expert researchers (FB, AA), who collated and arranged all the relevant information, bearing in mind this review’s main theme as expressed in the manuscript title.

## Vascular endothelial growth factor: biochemical properties and mechanisms of action

VEGF, also known as vascular permeability factor, was firstly described as an endothelial cell-specific mitogen [3]. This group of molecules belong to the cystine-knot superfamily of hormones and extracellular signalling molecules, covering several functions in vertebrates [4]. VEGF is a dimeric glycoprotein of ∼40 kDa and is fundamental in promoting growth metabolic cascades and angiogenesis during the development of the vertebrate retina [5,6]. VEGF family includes VEGF-A, VEGF-B, VEGF-C, VEGF-D, VEGF-E, VEGF-F, and PGF molecules. These mediators originate from the alternative splicing of a common source molecule and are further characterized by different isoforms [7]. Alternative splicing of the human VEGF-A gene provides at least six different isoforms, namely 121, 145, 165, 183, 189, and 206 [8]. VEGF-A_121_ and VEGF-A_165_ represent the most expressed forms in mammalians. With respect to VEGF-B, at least two isoforms are known, namely 167 and 186 [9]. On the other side, although largely studied in mouse models, less is known about the VEGF-C and VEGF-D isoforms [10–12]. VEGF-E is a molecule of ∼20 kDa, identified in the genome of Orf virus, a parapoxvirus that affects occasionally humans, generating lesions with angiogenesis [13], whereas VEGF-F is a toxin identified in the snake venom, having several similarities with the other VEGF isoforms [14].

All VEGF isoforms bind to different types of VEGF tyrosine kinase trans-membrane receptors; VEGFR-1/Flt-1 (fms-like tyrosine kinase) and VEGFR-2/KDR/Flk-1 (kinase insert domain containing receptor/fetal liver kinase) are mainly associated with angiogenesis [15], whereas Flt-3/Flk-2 and VEGFR-3/Flt-4 are involved in haematopoiesis and lymphangiogenesis [16].

VEGF-A isoforms exert the most powerful angiogenetic activity, also increasing vascular permeability, resulting in leakage of proteins and other molecules towards the blood vessels [17]. Furthermore, previous findings support the involvement of VEGF-A in neurotrophic and neuroprotective activities [18,19]. The angiogenic role of VEGF-B is quite unclear; this isoform seems to act more as a regulator of cells homeostasis and survival than as a active pro-angiogenic factor [20]. Conversely, VEGF-C has been found to play a major role in the lymphangiogenesis [21], although this isoform can also stimulate the growth of blood vessels [22]. Similarly, VEGF-D showed both angiogenic and lymphangiogenic activities [23,24].

VEGF is considered as the major factor involved in the angiogenesis of the human retina [25], although many other molecules and mediators are involved in angiogenic physiologic processes, turning out to be dysregulated in retinal vascular diseases [26,27]. However, because of the central role of VEGF-A, with respect to the other isoforms, many times researchers and clinicians refer to this subtype as simply VEGF [27].

Overall, different stimuli governed the production and release of VEGF. Its production is up-regulated by different isoforms of hypoxia inducible factor-1 (HIF-1), which are released to promote physiological angiogenesis, whereas other HIF isoforms act more as regulatory mediators of VEGF expression [28]. Moreover, VEGF levels can also be increased by insulin-like growth factor 1 (IGF-1), playing an important role in retinal angiogenesis [29]. In the human retina, VEGF is mainly produced by retinal pigmented epithelium (RPE) cells [30], astrocytes [31], Müller cells [32], endothelium and ganglion cells [33]. In retinal diseases, VEGF release is upregulated by increased hypoxia and oxidative stress [34,35]. Furthermore, the dysregulation of many other mediators is involved in the promotion of VEGF release, including erythropoietin (EPO) [36], angiopoietins 1 and 2 (Ang-1 and Ang-2), Tie2 receptor [15], platelet derived growth factor (PDGF) [37] and advanced glycation end-products [38]. In addition, VEGF isoforms may synergically act to further reinforce the cascades of events leading to VEGF metabolism alterations [39]. On the other hand, VEGF abnormal release is also influenced by the downregulation of inhibitory mediators. For example, neovascularization and exudation processes are enhanced by the alterations of the VEGF/pigment epithelium derived factor (PEDF) ratio. PEDF is more released by the peripheral RPE cells than macular RPE, acting as a major inhibitor of angiogenesis [40,41]. From this point of view, the peripheral ischaemia characterizing several retinal vascular diseases, including DR, might be the basis for VEGF/PEDF ratio dysregulation, thus providing a possible basis to explain why neovascularization and macular edoema mainly occur in the macular region, compared with retinal periphery.

## Vascular endothelial growth factor in diabetic retinopathy

**Figure 1.**
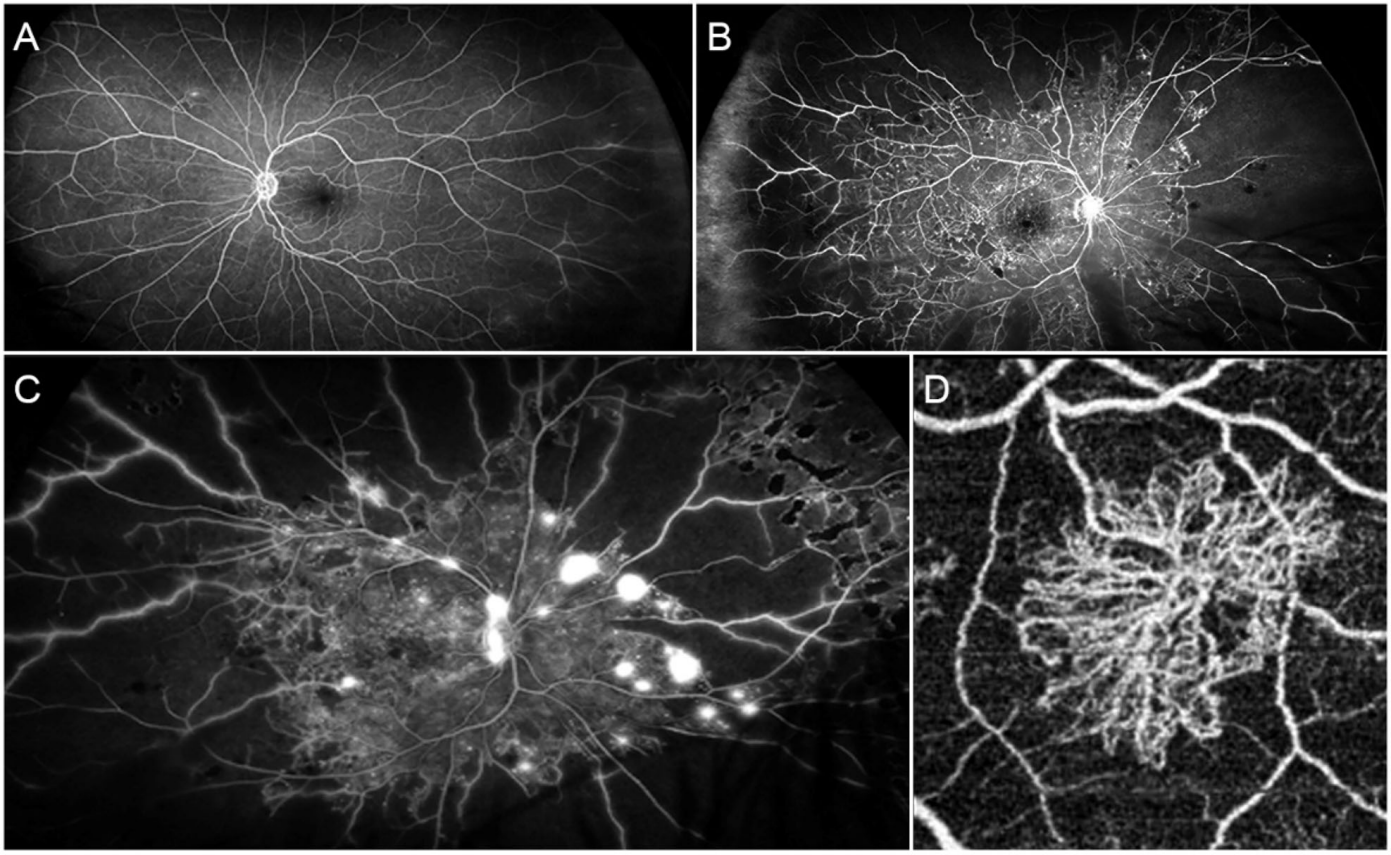
Angiographic findings in different stages of diabetic retinopathy. (A) Mild form of NPDR characterised by preserved posterior pole and minor capillary non-perfusion changes detected in the extreme periphery. (B) Advanced form of NPDR, showing extensive peripheral capillary non-perfusion and central involvement with several microaneurysms and macular edoema. (C) A case of PDR with extensive peripheral capillary non-perfusion and neovascularizations detected both at the level of the optic nerve head and the retinal periphery. (D) Optical coherence tomography angiography reconstruction of a PDR-related neovascularization.

DR is the most common microangiopathic complication of diabetes mellitus [2], affecting more than 100 million people worldwide and representing a major cause of visual impairment and legal blindness in developed countries [42]. DR is classically classified as non-proliferative (NPDR) and proliferative (PDR) (Figure 1). NPDR, further categorized in different stages, is characterized by several alterations including intraretinal haemorrhages, hard and soft exudates, cotton-wool spots, microaneurysms, venous calibre abnormalities, intraretinal microvascular anomalies and both macular and peripheral capillary nonperfusion; the onset of neovascularization characterize the progression towards PDR [2]. Most blindness cases are secondary to posterior PDR complications, such as vitreous haemorrhage and retinal detachment, whereas just 5% of blindness cases can be related to the onset of neovascular glaucoma [43]. The pathogenesis of DR is mainly characterized by the combined presence of neurovascular unit impairment, breakdown of the blood retinal barrier (BRB), inflammation, capillary non-perfusion/ischaemia, and neoangiogenesis [44]. All these anomalies lead to the increased production of angiogenic and inflammatory mediators. In this scenario, VEGF is undoubtedly a major pathogenic element characterizing DR and its complication. In DR, VEGF production is induced in response to ischaemic or hypoxic stimuli, causing several alterations at different levels. Indeed, VEGF increases the phosphorylation of tight junctions’ proteins, thus increasing retinal capillary permeability [45]. VEGF-related hyperpermeability is also caused by the induced modifications of several intercellular molecules, such as occludin, catenins and cadherins [46,47], by the increased transcytosis [48] and by the activated NOS-mediated mechanisms [49]. All these VEGF-related phenomena, mainly governed by VEGF-A isoforms, in combination with an extremely complex cascade of other events and mediators, lead to the onset and progression of a frequent DR-related complication, namely DME [50]. Furthermore, although few studies have been conducted, there are even growing evidences suggesting major roles of VEGF-B and PGF in DR pathogenesis. VEGF-B can promote neovascular phenomena and BRB breakdown towards non-inflammatory mechanisms, promoting potent survival stimuli on vascular and nonvascular cells [51]. Similarly, PGF acts as a powerful pro-angiogenic mediator, showing its serum and ocular concentrations strictly correlated with DR severity and risk of progression [51]. Hence, there is a consensus regarding the role of VEGF inhibition as a mandatory strategy for the management of DR [52].

With respect to the neovascular complication, the increased production and release of VEGF activates endothelial cell proliferation and migration, thus promoting neoangiogenesis [53]. Indeed, VEGF molecules activate two tyrosine kinase receptors, namely VEGFR-1 and VEGFR-2, stimulating endothelial proliferation, migration, and survival, thus promoting the progression to PDR [54]. Also in this case, VEGF acts as a major cause of DR-related neovascular process, although being just a part of a more complex pathogenic pathway. Indeed, it was demonstrated that angiopoietin system, over than regulating vascular integrity, it can promote and enhance the effect of VEGF neovascular stimulus [55]. Furthermore, neuropilin-1 (NRP1) was found to act as an isoform-specific receptor for VEGF_165_ and as a co-receptor of VEGF receptor 2 [56]. In addition, another system implicated on DR pathogenesis and on the enhancement of the neovascular stimulus provided by VEGF is the renin/angiotensin system. Indeed, the severity of DR, expressed both as the rate of progression of NPDR or the progression to PDR, were found strictly related to the activity of the renin/angiotensin system [57].

## Anti-vascular endothelial growth factor molecules

Based on all the above-described data, the main therapeutic target of anti-VEGF molecules regards the VEGF-A isoforms. It is worth of notice that endogenous anti-VEGF mechanisms already exist, although resulting impaired in retinal diseases such as DR. We have already described the role of VEGF/PEDF ratio dysregulation causing a reduced modulation of VEGF activity [40,41]. The physiologic anti-VEGF mechanisms represent a topic not deeply explored yet. Scant information came from animal models, reporting, for example, the anti-VEGF activity of VEGF_165b_ isoform [58]. More in detail, VEGF_165b_ seems to inhibit angiogenic stimuli induced by VEGF upregulation and hypoxia, also interfering with the migration and the proliferation of endothelial cells [58]. However, this topic would benefit from further dedicated studies to better define the endogenous anti-VEGF mechanisms occurring in animals and in human retina.

**Figure 2.**
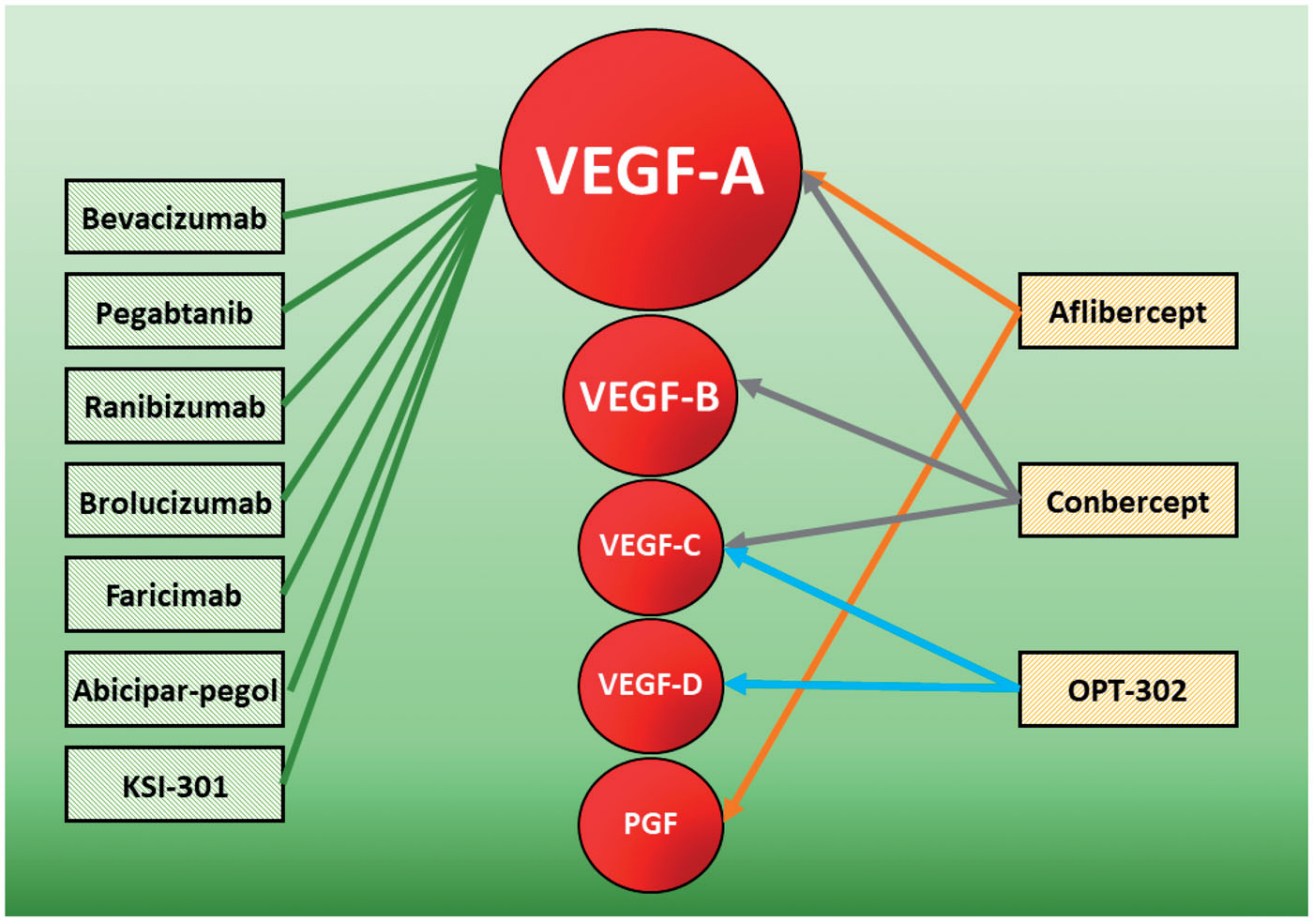
Main anti-VEGF molecules and respective VEGF isoforms targets.

With respect to anti-VEGF drugs, the available molecules currently include: Bevacizumab (Avastin^®^, Hoffmann-La Roche), Pegaptanib (Macugen, Eyetech/Pfizer), Ranibizumab (Lucentis^®^, Novartis Pharmaceuticals Canada Inc.), Aflibercept (Eylea^®^, BAYER Pharma AG, Germany), Conbercept (Chengdu Kanghong Biotech Company, China), Brolucizumab (Beovu^®^, Novartis Pharmaceuticals Canada Inc.), Abicipar-pegol (Allergan, Inc., Irvine, CA) and Faricimab (Hoffmann-La Roche) (Figure 2).

### Bevacizumab

Bevacizumab (Avastin^®^, Hoffmann-La Roche) is a fully humanized immunoglobulin G1 (IgG1) molecule of 148 kDa binding VEGF-A isoforms. This antibody was originally developed for cancer therapy [59]; its intravitreal use for retinal diseases is still considered as “off-label” treatment [60]. The mechanisms of action are quite simple; bevacizumab works as a pure anti-VEGF antibody, and its main effect is to block the neovascular stimulus and VEGF-induced increased vascular permeability [61]. Furthermore, bevacizumab can interact with HIF-1, thus interfering with its stimulating effect on VEGF production [62]. Although several studies described bevacizumab as an efficient and cost-effective treatment for retinal diseases [63–65], its usage is partially limited by its “off-label” classification. In 2007, the Diabetic Retinopathy Clinical Research Network (DRCR.net) reported the positive results regarding the employment of bevacizumab in DME on the basis of a phase II clinical trial [66]. Furthermore, the bevacizumab or laser therapy (BOLT) study reported the superiority of bevacizumab in the management of DME, with respect to laser approach [67,68]. Because of some doubts regarding the comparable profile of efficacy and safety of bevacizumab, the current recommendation of the European Society of Retina Specialists is to consider bevacizumab as a second choice, with respect to other anti-VEGF molecules [69].

### Pegaptanib

Pegaptanib (Macugen, Eyetech/Pfizer) was the first drug to obtain FDA approval for the treatment of retinal diseases through the intravitreal administration. This molecule is a pegylated‐aptamer binding preferentially the heparin-binding domain of VEGF165 isoform [70]. Although pegaptanib was found efficient in inhibiting the neovascularization process [71], its molecular features strongly limit its ability to block VEGF-related pathways, thus making it a poorly considered therapeutic choice.

### Ranibizumab

Ranibizumab (Lucentis^®^, Novartis Pharmaceuticals Canada Inc.) is a recombinant humanized immunoglobulin G1κ isotype monoclonal antibody fragment (Fab) of 48 kDa binding different VEGF-A isoforms and interfering with the interaction with VEGF receptors 1 and 2. The lack of fragment crystallizable (Fc) domain and the small molecule size might allowed to expand its affinity for more isoforms of VEGF-A (VEGF165, VEGF121, and VEGF110), increasing the penetration of the molecule within the retina and choroid [72,73]. Ranibizumab is characterized by only one binding site for VEGF; for this reason, two molecules of ranibizumab bind to one VEGF dimer [74]. This peculiar configuration allows the ranibizumab/VEGF-A complex having higher stability energy than bevacizumab [75] and greater molecular affinity to VEGF than bevacizumab and aflibercept [76]. Several clinical trials demonstrated high safety and efficacy of ranibizumab in the management of DME, exploring the usage of ranibizumab in different modalities and concentrations, as well as alone or in combination with laser, including READ-2 [77], RESOLVE [78], RESTORE [79], RISE and RIDE [80], LUCIDATE [81], REVEAL [82], RELIGHT [83], RETAIN [84] and READ-3 [85]. DRCR network conducted several multicenter clinical trials analyse similarities and differences of ranibizumab, with respect to other approaches, including other anti-VEGF molecules, corticosteroids, or laser, in the management of DR: Protocol S (ranibizumab vs. laser in PDR) [86,87], Protocol T (ranibizumab vs. aflibercept vs. bevacizumab in DME) [88] and Protocol I (fluocinolone acetonide vs. ranibizumab plus deferred laser in DME) [89] studies. Other meaningful clinical trials involving ranibizumab were: TREX-DME (ranibizumab “treat and extend” regimen with or without laser in DME) [90–92], ROTATE (ranibizumab in persistent DME after bevacizumab treatment) [93], RELATION (ranibizumab plus laser vs. laser alone in DR) [94] and REFINE (ranibizumab vs. laser in DME) [95] studies.

### Aflibercept

Aflibercept (Eylea^®^, BAYER Pharma AG, Germany) is a dimeric glycoprotein of 115 kDa, also known as VEGF Trap. This is obtained from the fusion of the first three Ig domains of VEGFR-1 and the Fc region of human IgG1 [96]. These biochemical properties provide high affinity for VEGF-A isoforms and PGF, as well as relative affinity for VEGF-B. Another version of the molecule, differing from aflibercept only for its excipients and the higher osmolarity, and displaying an almost identical biochemical profile, is Ziv-aflibercept (Zaltrap; Sanofi-Aventis and Regeneron Pharmaceuticals, Inc., Tarrytown, NY, USA) [97]. Although this molecule has been associated with promising effects in macular diseases, Ziv-aflibercept usage is still off-label [98]. The main trials dedicated on the assessment of the efficacy and safety of aflibercept in DR were VIVID-VISTA (aflibercept vs. laser in DME) [99,100], ENDURANCE (extension of VIVID-VISTA studies) [101], APOLLON (real-word data on aflibercept in DME) [102] and PANORAMA (aflibercept in NPDR) [103] studies. Furthermore, the DRCR network investigated the effect of aflibercept on DR progression, complications onset and outcome: Protocol V (aflibercept vs. laser vs. observation in DME) [102] and Protocol W (aflibercept vs sham in preventing vision-threatening complications in NPDR without DME) [103] studies.

### Conbercept

Conbercept (Chengdu Kanghong Biotech Company, China) is a molecule belonging to VEGF Trap family. It represents a full human DNA sequence of 143 kDa, obtained from the fusion of extracellular domain 2 of VEGFR-1 and extracellular domains 3 and 4 of VEGFR-2 with the Fc portion of human IgG1 [104,105]. The molecular characteristics of Conbercept are quite similar to aflibercept’s; this molecule differs for the presence of a portion dedicated to VEGFR-2, which was developed to potentially increase the efficacy and stability of Conbercept and to produce relative affinity for VEGF-C [104,105]. It turned out to be superior to laser treatment in DME, as assessed by SAILING study [106]. Further real-life reports described the efficacy and safety of Conbercept for DME management [107–110].

### Brolucizumab

Brolucizumab (Beovu^®^, Novartis Pharmaceuticals Canada Inc.) is a novel single-chain antibody fragment of 26 kDa, characterized by the absence of the Fc portion and developed to reduce molecule size and to improve the affinity for VEGF-A isoforms, compared with the other molecules [111,112]. Brolucizumab has been recently approved for the treatment of neovascular age-related macular degeneration, showing non-inferiority and higher penetrance within the retina and the choroid compared with the other anti-VEGF molecules [113,114]. With respect to DR, the ongoing KITE and KESTREL clinical trials reported preliminary positive results regarding the employment of brolucizumab in DME, compared with aflibercept [115], thus suggesting it will be approved for DR management soon.

### Abicipar-pegol

Abicipar-pegol (Allergan, Inc., Irvine, CA, USA) belongs to the family of designed ankyrin repeat proteins (DARPins) molecules, a class of proteins that can mimic antibodies showing high affinity for VEGF target [116]. In more details, abicipar-pegol is a recombinant protein of 34 kDa coupled to a polyethylene glycol fraction binding all VEGF-A isoforms [117]. Its affinity for VEGF-A turned out to be comparable to aflibercept’s but remarkably greater than bevacizumab’s and ranibizumab’s [118]. A phase II clinical trial was completed in 2015 about the employment of abicipar-pegol in DME (ClinicalTrials.gov ID: NCT02186119). However, further studies are warranted to support its role in DME management.

### Faricimab

Faricimab (Hoffmann-La Roche) is a novel molecule belonging to the DARPin family. This 150 kDa molecule can simultaneously and independently bind and neutralize both VEGF-A and Ang-2, the latter mechanism interfering with the Ang-1/Tie2 pathway [119]. The Ang-1/Tie2 pathway is a major pathogenic factor in the development of neovascularization and exudation. From this point of view, the multitargets profile of faricimab offers interesting new perspectives for the management of exudative retinal diseases. The phase 2 BOULEVARD trial provided prosing data regarding the employment of faricimab in DME, showing its superiority in terms of visual gain, compared with ranibizumab [120]. Furthermore, two Phase 3 clinical trials, RHINE and YOSEMITE (NCT03622593 and NCT03622580, respectively) are currently ongoing to compare the efficacy of faricimab to aflibercept [121]. However, also in this case, further studies are needed to draw more definite conclusions about the role of faricimab in DME management.

### Emerging anti-VEGF molecules

In this section, we would just mention two new anti-VEGF molecules currently under investigation. KSI-301 (KODIAK sciences, Palo Alto, CA), is a new generation antibody biopolymer conjugate, under study in a phase 1 b trial (NCT03790852) and in a DAZZLE phase 2 trial (NCT04049266), obtained from the combination of humanized anti-VEGF monoclonal antibody and a phosphorylcholine-based polymer, specifically designed to increase the duration of anti-VEGF activity. OPT-302 is a VEGF-C/D inhibitor, currently under investigation for exudative age-related macular degeneration in a Phase 2 b clinical trial (NCT03345082). In addition, a Phase 3 clinical trial (NCT03610646) is currently ongoing comparing aflibercept to intravitreal MYL-1701P, a recombinant fusion protein that is an aflibercept biosimilar [121].

All the above-mentioned clinical trials are extensively reported in Table 1.

**Table 1.** Main clinical trials dedicated on the assessment of anti-VEGF molecules in diabetic retinopathy. The order of appearance follows the year of publication.

N	Title	Acronym	Design	Number of Patients	Max duration	Drug	Year of publication	Main conclusions	Reference
1	Two-year outcomes of the ranibizumab for edoema of the mAcula in diabetes (READ-2) study	READ-2	Prospective, randomized, interventional, multicenter clinical trial	126	24 months	Ranibizumab	2010	Ranibizumab is effective in DME management with or without laser	77
2	Safety and efficacy of ranibizumab in diabetic macular edoema (RESOLVE Study): a 12-month, randomized, controlled, double-masked, multicenter phase II study	RESOLVE	Randomized, controlled, double-masked, multicenter phase II study	151	12 months	Ranibizumab	2010	Ranibizumab is effective in DME management	78
3	The RESTORE study: ranibizumab monotherapy or combined with laser versus laser monotherapy for diabetic macular edoema	RESTORE	Randomized, double-masked, multicenter, laser-controlled phase III study	345	12 months	Ranibizumab	2011	Ranibizumab with or without laser is superior to laser monotherapy for DME management	79
4	Ranibizumab for diabetic macular edoema: results from 2 phase III randomized trials: RISE and RIDE	RISE and RIDE	Two parallel, methodologically identical, phase III, multicenter, double-masked, sham injection-controlled, randomized studies	377 (RISE) and 382 (RIDE)	24 months	Ranibizumab	2012	Ranibizumab is effective in DME management	80
5	Randomized clinical trial evaluating intravitreal ranibizumab or saline for vitreous haemorrhage from proliferative diabetic retinopathy.	Protocol N	Phase 3, double-masked, randomized, multicenter clinical trial	261	4 months	Ranibizumab	2013	Ranibizumab is useful in vitrectomy setting of PDR	122
6	A randomized trial to assess functional and structural effects of ranibizumab versus laser in diabetic macular edoema (the LUCIDATE study).	LUCIDATE	Prospective, randomized, single-masked clinical trial	33	12 months	Ranibizumab	2014	Ranibizumab is effective in DME management	81
7	The REVEAL Study: Ranibizumab monotherapy or combined with laser versus laser monotherapy in Asian patients with diabetic macular edoema.	REVEAL	Randomized, double-masked, multicenter, laser-controlled, phase III study	396	12 months	Ranibizumab	2015	Ranibizumab with or without laser is superior to laser monotherapy for DME management	82
8	Ranibizumab 0.5 mg for diabetic macular edoema with bimonthly monitoring after a phase of initial treatment: 18-month, multicenter, phase IIIB RELIGHT Study.	RELIGHT	Phase IIIb, prospective, open-label, multicenter, single-arm study	109	18 months	Ranibizumab	2015	Ranibizumab is effective in DME management	83
9	Panretinal photocoagulation vs intravitreous Ranibizumab for proliferative diabetic retinopathy: a randomized clinical trial	Protocol S	Randomized, multicenter, clinical trial	394	Up to five years	Ranibizumab	2015-2018	Ranibizumab not inferior to PRP for PDR management	86,87
10	Intravitreal Aflibercept for diabetic macular edoema: 100-week results from the VISTA and VIVID studies.	VISTA and VIVID	Two similarly designed, double-masked, randomized, phase 3 trials, VISTA(DME) and VIVID(DME)	872	24 months	Aflibercept	2015	Aflibercept is effective in DME management	99,100
11	A study of Abicipar Pegol in patients with diabetic macular edoema (NCT02186119).	N/A	Randomized, multicenter study	151	12 months	Abicipar pegol	2015	Abicipar pegol non-inferior to ranibizumab for DME management	N/A
12	Ranibizumab 0.5 mg treat-and-extend regimen for diabetic macular oedema: the RETAIN study.	RETAIN	Single-masked, randomized study	372	24 months	Ranibizumab	2016	Ranibizumab is effective in DME management	84
13	Twenty-four-month outcomes of the Ranibizumab for edoema of the macula in diabetes – protocol 3 with high dose (READ-3) Study	READ-3	Randomized, controlled, double-masked (to the dose), interventional, multicenter clinical trial	152	24 months	Ranibizumab	2016	Ranibizumab is effective in DME management	85
14	Aflibercept, bevacizumab, or ranibizumab for diabetic macular edoema	Protocol T	Randomized, multicenter, clinical trial	660	24 months	Aflibercept, bevacizumab, and ranibizumab	2016	Aflibercept, bevacizumab, and ranibizumab non-inferiority study	88
15	Ranibizumab 0.3 mg for persistent diabetic macular edoema after recent, frequent, and chronic Bevacizumab: The ROTATE Trial.	ROTATE	Open-label, prospective study	30	12 months	Ranibizumab and bevacizumab	2016	Ranibizumab demonstrated improved outcome in patients with persistent DME following bevacizumab	93
16	Outcomes with as-needed Aflibercept and macular laser following the Phase III VISTA DME Trial: ENDURANCE 12-month extension study.	ENDURANCE	Phase IV, multicenter, open-label extension study (12-month extension of VISTA and VIVID)	60	36 months	Aflibercept	2017	Aflibercept with or without laser is superior to laser monotherapy for DME management	101
17	Clinical efficacy of intravitreal aflibercept versus panretinal photocoagulation for best corrected visual acuity in patients with proliferative diabetic retinopathy at 52 weeks (CLARITY): a multicentre, single-blinded, randomized, controlled, phase 2 b, non-inferiority trial.	CLARITY	Phase 2 b, single-blind, non-inferiority trial	232	12 months	Aflibercept	2017	Aflibercept is more effective than PRP for PDR management	123
18	Visual acuity outcomes in diabetic macular edoema with Fluocinolone Acetonide 0.2 μg/day versus Ranibizumab plus deferred laser (DRCR Protocol I).	Protocol I	Randomized, multicenter study	188	36 months	Ranibizumab and Fluocinolone Acetonide	2018	Fluocinolone Acetonide was comparable to ranibizumab plus deferred laser for DME management	89
19	The RELATION study: efficacy and safety of ranibizumab combined with laser photocoagulation treatment versus laser monotherapy in NPDR and PDR patients with diabetic macular oedema.	RELATION	Double-masked, multicentre phase IIIb study	128	12 months	Ranibizumab	2018	Ranibizumab with or without laser is superior to laser monotherapy for DME management	94
20	Ranibizumab plus panretinal photocoagulation versus panretinal photocoagulation alone for high-risk proliferative diabetic retinopathy (PROTEUS Study).	PROTEUS	Prospective, randomized, multicenter, open-label, phase II/III study	87	12 months	Ranibizumab	2018	Ranibizumab plus PRP superior to PRP monotherapy for DME management	124
21	Ranibizumab plus panretinal photocoagulation versus panretinal photocoagulation alone for high-risk proliferative diabetic retinopathy (PROTEUS Study).	PROTEUS	Prospective, randomized, multicenter, open-label, phase II/III study	87	12 months	Ranibizumab	2018	Ranibizumab plus laser superior than laser alone for PDR management	124
22	Efficacy and safety of ranibizumab 0.5 mg in Chinese patients with visual impairment due to diabetic macular edoema: results from the 12-month REFINE study.	REFINE	Phase III, double-masked, multicenter, laser-controlled study	384	12 months	Ranibizumab	2019	Ranibizumab with or without laser is superior to laser monotherapy for DME management	95
23	Effect of initial management with Aflibercept vs laser photocoagulation vs observation on vision loss among patients with diabetic macular edoema involving the centre of the macula and good visual acuity: a randomized clinical trial.	Protocol V	Randomized, multicenter study	702	24 months	Aflibercept	2019	Aflibercept is more effective than laser for DME management	102
24	Simultaneous inhibition of angiopoietin-2 and vascular endothelial growth factor-A with Faricimab in diabetic macular edoema: BOULEVARD Phase 2 randomized trial.	BOULEVARD	Prospective, randomized, active comparator-controlled, double-masked, multicenter, phase 2 study	229	6 months	Faricimab	2019	Faricimab was statistically superior than ranibizumab for DME management	120
25	Real-world outcomes following 12 months of intravitreal aflibercept monotherapy in patients with diabetic macular edoema in France: results from the APOLLON study.	APOLLON	Prospective, observational cohort study	147	12 months	Aflibercept	2020	Aflibercept is effective in DME management	125
26	A randomized, double-masked, multicenter, Phase III Study assessing the efficacy and safety of Brolucizumab versus Aflibercept in patients with visual impairment due to diabetic macular edoema (KITE).	KITE and KESTREL	Ongoing prospective, randomized, phase III clinical studies	361 (KITE) and 561 (KESTREL)	24 months	Brolucizumab and aflibercept	2020	Brolucizumab non-inferior to aflibercept for DME management	115
27	Long-term outcomes of treat-and-extend ranibizumab with and without navigated laser for diabetic macular oedema: TREX-DME 3-year results	TREX-DME	Multicenter, prospective, randomized clinical trial	116	36 months	Ranibizumab	2021	Ranibizumab is effective in DME management	90–92
28	Evaluation of intravitreal aflibercept for the treatment of severe nonproliferative diabetic retinopathy: results from the PANORAMA randomized clinical trial.	PANORAMA	Double-masked, multicenter, randomized clinical trial	402	24 months	Aflibercept	2021	Aflibercept is effective in DME management	126
29	Effect of intravitreous anti-vascular endothelial growth factor vs sham treatment for prevention of vision-threatening complications of diabetic retinopathy: the protocol W randomized clinical trial.	Protocol W	Randomized, multicenter study	328	24 months	Aflibercept	2021	Aflibercept is associated with lower progression to PDR than sham	103
30	Intravitreal conbercept for diabetic macular oedema: 2-year results from a randomized controlled trial and open-label extension study.	SAILING	Multicentre, randomized, double-masked, double-sham, parallel controlled, phase III trial (Sailing Study), followed by a 12-month open-label extension study	251/157	12 + 12 months	Conbercept	2021	Conbercept is more effective than laser for DME management	106
31	Effect of Aflibercept on diabetic retinopathy severity and visual function in the RECOVERY Study for proliferative diabetic retinopathy.	RECOVERY	Prospective, longitudinal, multicenter clinical trial	40	12 months	Aflibercept	2021	Aflibercept is associated with improvement of Diabetic Retinopathy Severity Scale for PDR eyes	127
32	A multi centre, randomized, double-masked, active-controlled, comparative clinical study to evaluate the efficacy and safety of MYL-1701P and Eylea® in subjects with diabetic macular edoema	N/A	Multi Centre, randomized, double-masked, active-controlled, comparative clinical study	355	12 months	MYL-1701P and aflibercept	N/A	Ongoing	121
33	A Phase III, multicenter, randomized, double-masked, active comparator-controlled study to evaluate the efficacy and safety of Faricimab (RO6867461) in patients with diabetic macular edoema (RHINE)	RHINE	Phase III, multicenter, randomized, double-masked, active comparator-controlled study	951	24 months	Faricimab and aflibercept	N/A	Ongoing	121
34	A Phase III, multicenter, randomized, double-masked, active comparator-controlled study to evaluate the efficacy and safety of Faricimab (RO6867461) in patients with diabetic macular edoema (YOSEMITE)	YOSEMITE	Phase III, multicenter, randomized, double-masked, active comparator-controlled study	940	24 months	Faricimab and aflibercept	N/A	Ongoing	121

## The role of anti-VEGF in DR-related retinal neovascularization

PDR is an extremely complex and vision-threating stage of DR. The management of this complicated form of DR has been mainly based on the employment of panretinal photocoagulation. The irreversible annihilation of peripheral ischaemic retina is associated with decreased production of VEGF and stabilization of the central retina, although having a remarkable impact on the visual field. Most of the above-described studies tried to assess the effect of anti-VEGF treatments on peripheral ischaemia and regression of the neovascularizations. The main question regards the possible replacement of panretinal photocoagulation with anti-VEGF injections alone. A Cochrane meta-analysis performed in 2014 reported low level of evidence regarding safety and efficacy of anti-VEGF in PDR, although the employment of intravitreal injections was associated with moderate reduction of the risk of intraocular bleeding [128]. A comparable low level of evidence was reported by another meta-analysis, although highlighting the higher morpho-functional outcome and benefits on vitrectomy setting obtained from anti-VEGF injections administered as adjuncts to panretinal photocoagulation [129]. The PROTEUS study assessed the role of adjunctive ranibizumab injections on panretinal photocoagulation vs laser treatment alone, reporting significant improvements of the visual acuity and fluorescein angiography features in PDR eyes undergoing combined approach [124]. DRCR Protocol S was a clinical trial specifically designed to compare panretinal photocoagulation to ranibizumab injections in PDR [86,87]. Overall, this study showed ranibizumab being non inferior to panretinal photocoagulation for PDR management. Eyes treated with laser showed higher risk of PDR progression, DME onset, and higher need of vitrectomy, compared with eyes treated by ranibizumab injections. Furthermore, Protocol S highlighted the importance of the starting PDR severity, associated with the presence of further complications, such as epiretinal membrane, neovascularization of the disc with neovascularization elsewhere, and vitreous haemorrhage on determining the achievable outcome [130]. The same attempt to compare panretinal photocoagulation with anti-VEGF injections was assessed by the CLARITY study, a phase 2 b, single-blind, noninferiority trial comparing aflibercept to panretinal photocoagulation in newly diagnosed or previously laser-treated active PDR [123]. Also in this case, the employment of anti-VEGF injections was associated with better outcome, compared with only laser-treated eyes, especially looking at neovascularization regression, visual acuity values and the need of vitrectomy. DRCR Protocol N was a phase 3, double-masked, randomized, multicenter clinical trial comparing intravitreal ranibizumab with intravitreal saline injections on vitrectomy rates for vitreous haemorrhage from PDR [122]. Because of the low overall rate of vitrectomies, the study could not detect differences in vitrectomy rates between groups. However, it showed how ranibizumab group showed better clinical outcome than eyes treated by saline injections. The non-inferiority of ranibizumab, compared with panretinal photocoagulation to manage PDR was furtherly confirmed by the five-year report of the DRCR network [87], which also reported relatively higher probability to develop DME and to have worse visual field in laser-treated group. Moreover, the recent RECOVERY study showed a statistically significant impact of anti-VEGF injections (in this case of aflibercept) on the improvement in DR severity progression registered after one year of follow-up in PDR eyes without DME [127]. Further recent meta-analysis studies provided even more support on the fact that, although not reaching enough level of statistical evidence, the use of anti-VEGF in PDR is associated with better visual outcome and less incidence of PDR-related complications, if compared with panretinal photocoagulation alone [131,132].

Overall considering all these findings, either anti-VEGF therapy or panretinal photocoagulation are feasible treatments for PDR patients. If panretinal photocoagulation was often criticized as inducing extensive loss of the visual field, the 5-year report of DRCR Protocol S highlighted a crucial point. Indeed, Maguire and colleagues [133] showed that, if the deterioration of the visual field was higher in the PDR group treated by laser during the first year, if compared with the eyes treated by anti-VEGF injections, the long-term visual field outcome was statistically similar between both groups at the end of the 5-year follow-up. These findings, showing similar long-term effect of laser and peripheral ischaemia on the visual field, further reinforced the role either of anti-VEGF and panretinal photocoagulation as effective treatments for PDR. As concluded by DRCR network, the therapeutic strategy for PDR should consider treatment recommendations, the relative advantages of each treatment approach and patients’ compliance with follow-up planning [134]. The other side of the medal is to always bear in mind the possible, although rare, risk of “anti-VEGF crunch syndrome”. This infrequent complication occurring in PDR is characterized by the progressive worsening of the fibrovascular tractional retinal detachment following anti-VEGF injections. This condition is still poorly defined, because of the very low incidence and the presence of many possible confounding factors, including panretinal photocoagulation. It might be determined by the fibrovascular regression induced by anti-VEGF injections, causing increased fibrosis leading to the worsening of the tractional component of the retinal detachment. As expected, the surgical approach and visual outcome are worse than PDR eyes not complicated by this occurrence [135]. The main interventional clinical trials specifically focussed on PDR are reported in Table 2.

**Table 2.** Interventional clinical trials specifically focussed on proliferative diabetic retinopathy.

N	Study ID	Title	Drug	Main Aim	Phase	Start Date	End Date	Status
1	NCT00131144	A randomized, controlled study on the efficacy and safety of octreotide acetate in microspheres in the therapy of patients with moderately severe or severe non-proliferate diabetic retinopathy (NPDR) or low risk proliferative diabetic retinopathy (PDR)	Octreotide acetate	To evaluate the efficacy of Octreotide acetate in PDR.	Phase III	1999	2005	Completed
2	NCT00130845	A randomized, controlled study on the efficacy and safety of octreotide acetate in microspheres in the therapy of patients with moderately severe or severe non-proliferate diabetic retinopathy (NPDR) or low risk proliferative diabetic retinopathy (PDR)	Octreotide acetate	To evaluate the efficacy of Octreotide acetate in PDR.	Phase III	2000	2005	Completed
3	NCT00170742	A randomized, open label, controlled study on the efficacy and safety of octreotide i.m. in patients with proliferative diabetic retinopathy (PDR) after start of laser coagulation	Octreotide, 30 mg i.m.	To evaluate monthly octreotide i.m. in comparison to no additional treatment in PDR after panretinal photocoagulation.	Phase III	2003	2006	Terminated
4	NCT00600262	Efficacy of intravitreal bevacizumab for severe nonproliferative and proliferative diabetic retinopathy.	Bevacizumab	To evaluate the 3-month efficacy of a single dose of intravitreal bevacizumab on the progression of severe NPDR and PDR.	Phase II/III	2005	2006	Completed
5	NCT00443521	Triamcinolone as adjunctive treatment to laser panretinal photocoagulation for proliferative diabetic retinopathy	Triamcinolone Acetonide	To evaluate the efficacy of adjuctive Triamcinolone Acetonide in PDR.	Phase II	2005	2006	Completed
6	NCT00248131	An open-label extension study to evaluate the long-term safety and tolerability of octreotide acetate in microspheres in the therapy of patients with moderately severe or severe non-proliferative diabetic retinopathy (NPDR) or low risk proliferative diabetic retinopathy (PDR)	Octreotide acetate	To evaluate the efficacy of Octreotide acetate in PDR.	Phase III	2005	2006	Terminated
7	NCT00248157	An open-label extension study to evaluate the long-term safety and tolerability of octreotide acetate in microspheres in the therapy of patients with moderately severe or severe non-proliferative diabetic retinopathy (NPDR) or low risk proliferative diabetic retinopathy (PDR)	Octreotide acetate	To evaluate the efficacy of Octreotide acetate in PDR.	Phase III	2005	2006	Terminated
8	NCT00423059	Histologic changes of fibrovascular membrane associated with proliferative diabetic retinopathy after intravitreal Bevacizumab (Avastin®).	Bevacizumab	To evaluate the effect of the intravitreal bevacizumab on the fibrovascular membrane associated with PDR by objective histologic evaluation in eyes underwent vitrectomy.	N/A	2006	2007	Completed
9	NCT00446381	Effect of Macugen (Pegaptanib) on surgical outcomes and growth factors including vascular endothelial growth factor (VEGF) levels in patients with proliferative diabetic retinopathy (PDR) and clinically significant diabetic macular edoema (CSDME).	Pegaptanib sodium	To quantify the reduction of intravitreal VEGF 165 levels in patients following intravitreal Macugen injection pre-operatively.	N/A	2006	2008	Completed
10	NCT00656435	Bevacizumab pre-treatment and long acting gas infusion on the vitreous clear-up after diabetic vitrectomy	Bevacizumab	To evaluate the efficacy of bevacizumab before vitrectomy in PDR.	Phase III	2006	2008	Completed
11	NCT01041690	Bevacizumab (Avastin) as an adjunct to vitrectomy in the management of severe proliferative diabetic retinopathy: a prospective case series.	Bevacizumab	To evaluate the role of preoperative intravitreal bevacizumab as an adjunct to vitrectomy in the management of PDR.	N/A	2007	2008	Completed
12	NCT00596505	Intravitreal Bevacizumab (Avastin) pre-treatment for reducing intraoperative and postoperative preretinal haemorrhage in primary diabetic vitrectomy with silicone oil infusion.	Bevacizumab	To evaluate the effect of pre-operative becacizumab in PDR eyes undergoing vitrectomy.	N/A	2007	2007	Completed
13	NCT00548197	Preoperative injection of Bevacizumab prior to vitreoretinal surgery in diabetic tractional retinal detachment.	Bevacizumab	To evaluate the preoperative injection of bevacizumab on PDR eyes undergoing vitrectomy.	Phase I	2007	2008	Completed
14	NCT00445003	Intravitreal Ranibizumab or triamcinolone acetonide as adjunctive treatment to panretinal photocoagulation for proliferative diabetic retinopathy	Ranibizumab, Triamcinolone Acetonide	To evaluate the efficacy of intravitreal triamcinolone or intravitreal ranibizumab in preventing loss of vision caused by panretinal photocoagulation treatment in PDR.	Phase III	2007	2010	Completed
15	NCT00668785	Intravitreal Ranibizumab to treat macular edoema after panretinal photocoagulation (Phase II)	Ranibizumab	To evaluate the efficacy of ranibizumab in PDR.	Phase II	2007	2012	Terminated
16	NCT00606138	Investigation of Ranibizumab for the treatment of persistent diabetic neovascularization as assessed by super wide-field angiography (Optos).	Ranibizumab	To evaluate the efficacy of ranibizumab versus additional panretinal photocoagulation on diabetic neovascularization that is persistent despite previous treatment with panretinal photocoagulation.	Phase I/II	2008	2010	Completed
17	NCT00545870	A randomized, double-masked study with intraocular Bevacizumab compared with intravitreal Ranibizumab in patients with persistent diabetic macular edoema or persistent active neovascularisation following lasercoagulation	Bevacizumab, ranibizumab	To evaluate safety and efficacy of ranibizumab vs bevacizumab following panretinal photocoagulation in PDR.	Phase III	2008	2013	Completed
18	NCT00511875	Evaluation of effect of doxycycline verses placebo on diabetic retinopathy progression and retinal function in patients with severe non-proliferative or mild or moderate (non-high-risk) proliferative diabetic retinopathy.	Doxycycline monohydrate	To evaluate the effect of doxycycline monohydrate in DR and PDR.	Phase II	2008	2012	Completed
19	NCT00745498	Efficacy study of pre- and intra-operative intravitreal Bevacizumab injection on postoperative vitreous haemorrhage after diabetic vitrectomy.	Bevacizumab	To evaluate the effect of pre- and intra-operative bevacizumab injection on PDR eyes undergoing vitrectomy.	N/A	2008	2010	Completed
20	NCT01102946	Panretinal photocoagulation versus panretinal photocoagulation plus intravitreous Ranibizumab for high risk proliferative diabetic retinopathy.	Ranibizumab	To evaluate safety and efficacy of ranibizumab + panretinal photocoagulation in the regression of retinal neovascularization in PDR.	Phase II	2009	2011	Completed
21	NCT00907114	Efficacy and safety of topic Ketorolac to treat centre point thickness secondary to panphotocoagulation in proliferative diabetic retinopathy.	Ketorolac tromethamine	To evaluate the efficacy and safety of topic ketorolac in treatment for centre point thickness secondary to panphotocoagulation in PDR.	Phase II	2009	2015	Completed
22	NCT01270542	Effect of pre-operative adjunctive anti-VEGF on growth factors in severe proliferative diabetic retinopathy requiring surgical management	Bevacizumab	To evaluate the efficacy of bevacizumab before vitrectomy in PDR.	N/A	2009	2012	Completed
23	NCT01280929	Prospective, randomized, multicenter, open label, Phase II Study to access efficacy and safety of Lucentis® monotherapy (Ranibizumab 0.5 mg intravitreal injections) compared with Lucentis® Plus panretinal photocoagulation (PRP) and PRP (monotherapy) in the treatment of patients with high risk proliferative. diabetic retinopathy	Ranibizumab	To evaluate safety and to compare the efficacy of ranibizumab + panretinal photocoagulation vs panretinal photocoagulation alone in the regression of retinal neovascularization in PDR.	Phase II	2010	2013	Completed
24	NCT01281098	Prospective, randomized, open label, Phase II Study to assess efficacy and safety of Macugen® (Pegaptanib 0.3 mg intravitreal injections) plus panretinal photocoagulation (PRP) and PRP (monotherapy) in the treatment of patients with high risk proliferative diabetic retinopathy (PDR).	Pegaptanib sodium	To evaluate the safety and determine the efficacy of panretinal photocoagulation monotherapy or combination therapy (pegaptanib 0.3 mg plus panretinal photocoagulation) in PDR.	Phase II	2010	2013	Completed
25	NCT00996437	An evaluation of intravitreal Ranibizumab for vitreous haemorrhage due to proliferative diabetic retinopathy.	Ranibizumab	To evaluate the efficacy of intravitreal ranibizumab in vitrectomy setting in PDR.	Phase II	2010	2013	Completed
26	NCT01213888	Trientine Hydrochloride for the prevention of macular edoema associated with pan-retinal photocoagulation for severe non-proliferative and proliferative diabetic retinopathy	Trientine Hydrochloride	To evaluate the efficacy of Trientine Hydrochloride in PDR.	N/A	2010	2013	Terminated
27	NCT01487070	A single-centre trial of intravitreous injections of Macugen (Pegaptanib Sodium) given at least 7 days before vitrectomy secondary to tractional retinal detachment in proliferative diabetic retinopathy	Pegaptanib sodium	To evaluate the safety and efficacy of intravitreal injections of pegaptanib sodium in PDR undergoing vitrectomy.	Phase I	2011	2011	Completed
28	NCT01746563	Intravitreal Ranibizumab combined with panretinal photocoagulation in patients with treatment-naive proliferative diabetic retinopathy	Ranibizumab	To evaluate the efficacy of ranibizumab plus panretinal photocoagulation versus panretinal photocoagulation alone in PDR.	Phase I	2011	2012	Completed
29	NCT01594281	Multicenter randomized open-label three-arms controlled 12 months clinical proof of concept study to evaluate efficacy and safety of Ranibizumab alone or in combination with laser photocoagulation vs. laser photocoagulation alone in proliferative diabetic retinopathy.	Ranibizumab	To evaluate safety and to compare the efficacy of ranibizumab with or without panretinal photocoagulation vs panretinal photocoagulation alone in the regression of retinal neovascularization in PDR.	Phase II	2012	2017	Completed
30	NCT01589029	A Pilot Study on the effects of ILARIS® on patients with proliferative diabetic retinopathy (PDRP).	Canakimumab	To evaluate the efficacy and safety of Canakinumab (ILARIS®) in PDR.	Phase I	2012	2014	Terminated (primary endpoint not met)
31	NCT01535495	Propranolol for diabetic retinopathy.	Propanolol	To evaluate oral beta antagonist propranolol efficacy in inducing regression of retinal neovascularization in PDR.	Phase I	2012	2015	Completed
32	NCT02816073	Single-session pattern scanning laser pan-retinal photocoagulation in proliferative diabetic retinopathy - a randomized study	Laser	To evaluate the safety and efficacy of single-session panretinal photocoagulation using Pattern Scan Laser (PASCAL) in PDR.	N/A	2012	2016	Completed
33	NCT01489189	Prompt panretinal photocoagulation versus intravitreal Ranibizumab with deferred panretinal photocoagulation for proliferative diabetic retinopathy.	Ranibizumab	To evaluate the effect of prompt panretinal photocoagulation vs intravitreal ranibizumab with deferred panretinal photocoagulation in PDR.	Phase III	2012	2018	Completed
34	NCT01589718	A Phase III randomized 1:1, masked, study of the safety, tolerability, and efficacy of intravitreal pre-op 0.3 mg Pegaptanib Sodium versus Sham, for adjuvant management of TRD and Vit Hem associated with PDR.	Pegaptanib sodium	To evaluate preoperative the efficacy of pegaptanib sodium in improving vitreous haemorrhage prior to vitrectomy in PDR.	Phase III	2012	2014	Withdrawn (No patients were enrolled)
35	NCT01854593	Prospective randomized controlled study of intravitreal injection of 0.16 mg Bevacizumab one day before surgery for proliferative diabetic retinopathy.	Bevacizumab	To evaluate the efficacy of bevacizumab before vitrectomy in PDR.	Phase IV	2012	2014	Completed
36	NCT01627977	A descriptive study to evaluate the effectiveness of the dye compound of the combination of Lutein, Zeaxanthin and brilliant blue in Chromovitrectomy	Dye of Lutein, Zeaxanthin and Brilliant Blue	To evaluate the efficacy of Lutein, Zeaxanthin and Brilliant Blue in vitrectomy setting in PDR.	Phase III	2012	2014	Completed
37	NCT01552408	A Phase I/II, randomized, study for diabetic macular edoema using 0.3 mg Ranibizumab combined with targeted PRP monthly for 4 months, then PRN vs. 0.3 mg Ranibizumab 4 months monotherapy, then as needed(DME-AntiVEgf) DAVE	Ranibizumab	To evaluate the efficacy of ranibizumab combined with panretinal photocoagulation in PDR.	Phase I/II	2012	2017	Completed
38	NCT01769183	Topical Squalamine in the treatment of retinal neovascularization from proliferative diabetic retinopathy.	Squalamine Lactate ophthalmic solution 0.2%	To determine the efficacy of topical Squalamine Lactate Ophthalmic Solution 0.2% in the treatment of retinal neovascularization in PDR.	Phase II	2013	2014	Completed
39	NCT01908816	An open-label extended clinical protocol of Ranibizumab to evaluate safety and efficacy in rare VEGF driven ocular diseases.	Ranibizumab	To evaluate the safety and the efficacy of ranibizumab in DR.	Phase III	2013	2016	Completed
40	NCT01813773	Treatment with intravitreal Aflibercept injection for proliferative diabetic retinopathy, The A.C.T Study.	Aflibercept	To evaluate the safety of intravitreal aflibercept injection in the treatment of PDR.	Phase II/III	2013	2016	Completed
41	NCT01805297	Intravitreal Aflibercept injection as a surgical adjuvant in severe proliferative diabetic retinopathy	Aflibercept	To evaluate the effocacy of aflibercept in vitrectomy setting in PDR.	Phase II	2013	2015	Completed
42	NCT01869933	An open label Phase I placebo controlled, dose escalation study assessing the ocular and systemic safety and tolerability of OC-10X	OC-10X	To evaluate safety and tolerability of OC-10X in PDR.	Phase I	2013	2013	Completed
43	NCT01941329	Prospective, randomized, multicentre, open-label, Phase II/III Study to assess efficacy and safety of Ranibizumab 0.5 mg intravitreal injections plus panretinal photocoagulation (PRP) versus PRP in monotherapy in the treatment of subjects with high risk proliferative diabetic retinopathy. (PROTEUS)	Ranibizumab	To evaluate safety and to compare the efficacy of ranibizumab + panretinal photocoagulation vs panretinal photocoagulation alone in the regression of retinal neovascularization in PDR.	Phase II/III	2014	2017	Completed
44	NCT02735369	A Phase II, randomized, placebo-controlled, study assessing efficacy and safety of OC-10X ophthalmic suspension in the treatment of proliferative diabetic retinopathy	2% OC-10X	To evaluate the efficacy and safety of topical OC-10X Ophthalmic Suspension in PDR.	Phase II	2014	2016	Terminated
45	NCT03904056	ETDRS panretinal photocoagulation (PRP) combined with intravitreal Ranibizumab (IVR) versus retinal photocoagulation targeted to ischaemic retina combined with IVR for the treatment of proliferative diabetic retinopathy.	Laser, ranibizumab	To compare panretinal photocoagulation combined with intravitreal injection of ranibizumab and retinal photocoagulation targeted to ischaemic retina combined with intravitreal injections of ranibizumab in PDR.	N/A	2014	2017	Completed
46	NCT02857491	Comparison of intravitreal injection of Ranibizumab versus Sham Injection before vitrectomy in patients with proliferative diabetic retinopathy: a single-centre, prospective double-blinded randomized controlled trial.	Ranibizumab	To evaluate the effect of pre-operative injection of ranibizumab on peri-operative hemorrahge related complications in PDR.	N/A	2014	2017	Completed
47	NCT02475109	A Phase 1 open-label, single-centre study to evaluate the safety and tolerability of topical ocular PAN-90806 in patients with proliferative diabetic retinopathy (PDR)	PAN-90806 Ophthalmic Solution	To evaluate the safety and tolerability of topical ocular PAN-90806 in PDR.	Phase I	2015	2016	Completed
48	NCT02447185	25-Gauge vitrectomy with Ranibizumab or Triamcinolone Acetonide on proliferative diabetic retinopathy in China: a randomized, Single Blind Trial.	Ranibizumab, Triamcinolone Acetonide	To evaluate the efficacy of intravitreal triamcinolone or intravitreal ranibizumab in vitrectomy setting in PDR.	Phase III	2015	2021	Recruiting
49	NCT02590094	Comparison of interval variation and dosage in preoperative Bevacizumab and Ziv-Aflibercept administration in proliferative diabetic retinopathy undergoing vitrectomy	Bevacizumab, ziv-aflibercept	Bevacizumab versus ziv-aflibercept in PDR.	N/A	2015	2023	Recruiting
50	NCT02634333	Intravitreous anti-vascular endothelial growth factor treatment for prevention of vision threatening diabetic retinopathy in eyes at high risk.	Aflibercept	To evaluate the efficacy and safety of intravitreous aflibercept injections versus sham injections (observation) for prevention of PDR.	Phase III	2016	2022	Active, not recruiting
51	NCT02816710	Different Conbercept injection methods in treatment of severe proliferative diabetic retinopathy.	Conbercept	To evaluate the efficacy of pre-, intra- or post-operative conbercept in PDR eyes undergoing vitrectomy.	Phase IV	2016	2017	Completed
52	NCT02863354	Intravitreal Aflibercept for retinal non-perfusion in proliferative diabetic retinopathy.	Aflibercept	To evaluate the safety and tolerability of 2 mg intravitreal aflibercept injections in PDR.	Phase II	2016	2019	Completed
53	NCT02705274	Panretinal photocoagulation versus intravitreal Bevacizumab for proliferative diabetic retinopathy.	Bevacizumab	To evaluate the effect of bevacizumab and panretinal photocoagulation administered on the basis of DRCR Protocol S data in PDR.	Phase II/III	2016	2017	Completed
54	NCT02753400	A multicenter, randomized, double-masked, placebo-controlled, pilot study to evaluate effects of Emixustat Hydrochloride on aqueous humour biomarkers associated with proliferative diabetic retinopathy.	Emixustat hydrochloride	To evaluate the effects of emixustat in PDR.	Phase II	2016	2017	Completed
55	NCT02858076	Intravitreous anti-VEGF vs. prompt vitrectomy for vitreous haemorrhage from proliferative diabetic retinopathy.	Aflibercept	To evaluate the efficacy of aflibercept vs. laser in PDR.	Phase II	2016	2020	Completed
56	NCT03426540	Safety and efficacy of intravitreal Conbercept injection after vitrectomy for the treatment of early proliferative diabetic retinopathy.	Conbercept	To evaluate safety and efficacy of intravitreal conbercept injection after vitrectomy in PDR.	Phase I	2017	2019	Completed
57	NCT03113006	The individually-marked panretinal laser photocoagulation for proliferative diabetic retinopathy study: IMPETUS 2018 – TREAT.	Laser	To evaluate laser treatment protocol in PDR.	N/A	2017	2019	Completed
58	NCT02151695	Phase 2 Study of Safety and Efficacy of Aflibercept in Proliferative Diabetic Retinopathy.	Aflibercept	To evaluate the efficacy and the safety of aflibercept intravitreal injections compared to panretinal photocoagulation in PDR.	Phase II	2018	2020	Completed
59	NCT03633266	Feasibility study of anti-VEGF instead of intraoperative PRP in proliferative diabetic retinopathy.	Anti-VEGF	To evaluate the efficacy and safety of vitreoretinal surgery combined with anti-VEGF therapy in PDR.	N/A	2018	2022	Not yet recruiting
60	NCT02911311	Conbercept vs. panretinal photocoagulation for the management of proliferative diabetic retinopathy.	Conbercept	To evaluate the efficacy and safety between intravitreal injection of conbercept and panretinal photocoagulation in PDR.	N/A	2019	2022	Enrolling by invitation
61	NCT04278417	A 96-week, two-arm, randomized, single-masked, multi-centre, Phase III Study assessing the efficacy and safety of Brolucizumab 6 mg compared to panretinal photocoagulation laser in patients with proliferative diabetic retinopathy	Brolucizumab	To evaluate the efficacy and safety of brolucizumab compared to panretinal photocoagulation in PDR.	Phase III	2020	2024	Recruiting
62	NCT04800679	Combination therapy for PDR.	Bevacizumab	To evaluate the efficacy of combination laser and bevacizumab therapy in PDR.	Phase II	2020	2021	Recruiting
63	NCT04464694	Prospective, single-blind, randomized, controlled, multi-centre study to evaluate the benefit of Ranibizumab as an adjunctive therapy to vitrectomy for patients with proliferative diabetic retinopathy combined with diabetic macular oedema.	Ranibizumab	To evaluate ranibizumab's benefit on prevention of early postoperative vitreous haemorrhage in PDR.	Phase IV	2020	2022	Not yet recruiting
64	NCT04424290	A first-in human trial to study safety and tolerability of single rising intravitreal dOses (open label, non-randomized, uncontrolled) and in addition the early biological response of multiple intravitreal dosing (single-masked, randomized, Sham-controlled) of BI 764524 in panretinaL photocoagulation (PRP) treated proliferative diabetic retinopathy (PDR) patients with diabetic macular ischaemia (DMI) - the HORNBILL Study	BI 764524	To evaluate the efficacy of BI 764524 in PDR	Phase I/II	2020	2022	Recruiting
65	NCT04674254	Macular perfusion changes in proliferative diabetic retinopathy following anti-VEGF therapy versus targeted and pan-retinal photocoagulation using optical coherence tomography angiography.	Bevacizumab	To evaluate the effect of bevacizumab, targeted retinal photocoagulation and standard retinal panphotocoagulation on macular perfusion in PDR.	Phase IV	2021	2023	Recruiting
66	NCT04661358	A randomized clinical trial evaluating fenofibrate for prevention of diabetic retinopathy worsening.	Fenofibrate 160 mg	To evaluate the effect of fenofibrate compared with placebo for prevention of DR worsening.	Phase III	2021	2027	Recruiting
67	NCT04692688	Randomized, placebo-controlled, double-masked study of the safety and efficacy of orally administered APX3330 in subjects with moderately severe to severe non-proliferative diabetic retinopathy and mild proliferative diabetic retinopathy	APX3330	To evaluate the efficacy of APX3330 in PDR.	Phase II	2021	2022	Recruiting

*Source*: https://clinicaltrials.gov/. The order of appearance follows the start date.

## Final remarks and conclusions

Neovascular complication may have a remarkable negative impact on patients’ management and visual outcome in DR and is characterized by increased production of VEGF as a major causative factor. In the present review, we provided an overall description of the use of anti-VEGF molecules for the management of PDR, focussing on the current drugs, the molecules under investigation and the possible targets ruling the neovascular process and the development of further complications. It is unquestionable that the introduction of anti-VEGF therapy radically changed the management and prognosis of all the stages of DR, including PDR. Before the anti-VEGF era, the only therapeutic approach was represented by laser, performed on retinal periphery, through panretinal photocoagulation, or at the posterior pole, through focal/grid treatments. Laser approaches turned out to be useful in managing DME and neovascular progression, although the irreversible demolitive effect on retinal structures had a negative impact on the morpho-functional status of diabetic eyes. As highlighted by the present review, a big debate is nowadays present in the literature regarding the comparison between anti-VEGF and laser approaches, and the combined use of both treatments. Most of the current data do not provide enough level of evidence to draw definite conclusions. Through the many studies conducted, the DRCR network offered a strong contribution in the clinical and therapeutic management of DR, highlighting the importance of promptly and frequently repeated intravitreal injections. The overall feel is that laser approach might be avoided when a perfectly planned anti-VEGF therapeutic strategy can be adopted. However, we must bear in mind that the real-life situation is quite different from clinical trials settings. Probably, there is no absolute winning therapeutic choice for managing DR, but the choice of the treatments and of the follow-up timeline must be planned based on personalized strategies designed on patients’ characteristics. The clinical status, including glycemic control and presence of comorbidities, patient’s self-sufficiency, compliance with the frequency of follow-up visits and treatments represent key aspects ruling the overall clinical and ophthalmologic management of the DR patient. The future development of longer duration anti-VEGF treatments and of even more optimized molecules will have a remarkable impact on the feasibility and sustainability of DR patients care for the hospitals and the public health systems, and probably will modify the current indications to laser approaches. According to the EURETINA guidelines [67], anti-VEGF molecules represent the treatment of choice for most of DR patients, because of the high efficacy and safety profiles, and the feasible management. Possible contra-indications regard those patients characterized by high cardiovascular risks, where other approaches, such as corticosteroids [135–138], should be preferred. Laser approaches still maintain a role for diabetic eyes characterized by extremely severe form of DR and for those patients not guaranteeing adequate compliance to intravitreal treatments strategies.

## Data Availability

Data are available after a formal request to the corresponding author.
